# Urinary Tract Infection and Antimicrobial Susceptibility of Bacterial Isolates in Saint Joseph Kitgum Hospital, Kitgum, Uganda

**DOI:** 10.3390/antibiotics11040504

**Published:** 2022-04-11

**Authors:** Félix Carrasco Calzada, John Jairo Aguilera-Correa, Juan Cuadros González, Jaime Esteban Moreno, David Roca Biosca, Ramón Pérez-Tanoira

**Affiliations:** 1 Health Sciences Department, Faculty of Med, Universidad de Alcalá de Henares, 28805 Alcalá de Henares, Spain; juan.cuadros@uah.es; 2 Clinical Microbiology Department, Hospital Universitario Príncipe de Asturias, 28805 Alcalá de Henares, Spain; 3 IIS-Fundación Jiménez Díaz, 28007 Madrid, Spain; john.aguilera@fjd.es (J.J.A.-C.); jesteban@fjd.es (J.E.M.); 4 Fundación El Alto, 12500 Vinaroz, Spain; davidrocabiosca@yahoo.es

**Keywords:** antibiotic sensitivity test, prevalence, urinary tract infection, developing countries, Uganda

## Abstract

A cross-sectional study of microorganisms isolated from mid-stream urine samples obtained from 139 patients with suspected urinary tract infection (UTI) who presented leukocyturia was conducted from April to June 2019 at Saint Joseph Kitgum Hospital (Uganda). All microorganisms were identified by MALDI-TOF mass spectrometry in a laboratory in Spain. Antimicrobial susceptibility was determined on site using the disc diffusion method (Kirby–Bauer test) and these results were subsequently compared with those obtained in Spain using the Becton Dickinson Phoenix M50 device. The overall prevalence of UTI with bacterial growth was 64.0% (*n* = 89) (95% CI, 56.1–72.0), and 11 presented mixed infection. As a result, 100 microorganisms were isolated. The most common uropathogens were *Enterococcus* spp. (57%) and *Escherichia coli* (28%). Nitrofurantoin was the most effective drug (81.7% in Gram-positive and 87.3% in Gram-negative bacteria), followed by imipenem (94.2% and 74.5%, respectively). The highest resistance rates were observed for amoxicillin and ciprofloxacin (66.2% and 44.6%, respectively). Given the increasing trend toward antibiotic resistance, there is a need for bacteriological cultures and continuous surveillance of uropathogen antibiotic susceptibility. Use of amoxicillin and ciprofloxacin as empirical treatments for UTIs should be discontinued in Uganda. The findings of this study may be useful for clinicians, as they may improve empirical treatment.

## 1. Introduction

Urinary tract infections (UTIs), which occur frequently in both hospital and community settings, are some of the most common bacterial infections. Symptoms associated with UTIs are associated with widespread use of broad-spectrum antibiotics due to self-medication or inappropriate empirical treatment of patients who present to clinics or emergency-care facilities, which leads to rapid spreading of multidrug-resistant bacteria [1,2].

Most UTIs occur in women with no underlying diseases and no functional or structural abnormalities of the urinary tract, and as a result, these are considered uncomplicated UTIs [3]. Other forms of UTI presentation include asymptomatic bacteriuria (AB) and complicated UTIs [3]. Complicated UTIs are a heterogeneous group of conditions that increase the risk of infection or treatment failure. AB refers to the presence of substantial quantitative counts of bacteria in a urine sample; as this condition is benign, it most commonly goes undetected and does not require antibiotic treatment in healthy, non-pregnant women. However, in 25–30% of pregnant women with AB, there is a risk of developing acute pyelonephritis during labour, which can cause several adverse events in newborns such as preterm rupture of the membrane, chorioamnionitis, preterm birth, and neonatal sepsis [4,5]. Inadequate follow-up and study of these conditions can generate a high rate of antimicrobial resistance, which in developing countries is aggravated by limited access to therapeutic alternatives [1].

In recent years, there have been substantial changes in the susceptibility patterns of the main urinary pathogens, including a progressive increase in infections caused by extended-spectrum beta-lactamase (ESBL)-producing enterobacteria or even carbapenemase-producing bacteria [6,7], leading to changes in the empirical treatment of these infections. However, the increase in resistance must bring about changes not only in empirical therapy, but must also lead to efforts that encourage rational use of antibiotics [2].

The problem of antimicrobial resistance is accentuated in rural hospitals located in developing countries due first to the low availability of antibiotics and second to the lack of a robust etiological diagnosis, exacerbated in many cases by the absence of antimicrobial sensitivity studies [8]. Together, these factors lead to excessive, uncontrolled use of broad-spectrum antibiotics, which negatively impacts the economy and health of these societies due, for example, to prolonged hospital stays and lack of treatment efficacy [1,8]. Accurate microbiological diagnosis of infectious diseases and the administration of adequate treatment are limited by the lack of material resources and laboratory personnel trained in these techniques [8,9].

In developing countries, scant access to quality-assured antibiotics drives people to purchase antibiotics without a prescription on the informal market, and currently these patients are not accounted for under surveillance programs. The unregulated sale of antibiotics contributes to the overuse and misuse of these medicines [1]. Nitrofurantoin has been recently instituted as the treatment of choice for UTIs, while ciprofloxacin and amoxicillin are used as second-line empirical treatment [10]. Although recent studies evidence the high effectiveness of nitrofurantoin [8], second-line treatments have not shown the same results, and more exhaustive research is necessary [8,11,12]. Finally, it is noteworthy that little research has examined the prevalence of common pathogens and their susceptibility to antibiotics in rural Uganda.

In this study, we aimed to determine the main etiologic agents in UTIs in North Uganda and establish the antibiotic susceptibility profiles of these pathogens. Our hypothesis is that by conducting a microbiological study of the bacteria involved in diagnostic urine infections in a rural area of Uganda, where the antimicrobial susceptibility of the pathogens involved is unknown, we will be able to provide effective antibiotic treatment.

## 2. Results

Between April and June 2019, a total of 1004 urine samples from patients with suspected UTI were analysed. Of the 139 (13.8%) samples with leukocyturia, 89 showed bacterial growth.

A total of 89 patients had a positive culture, with a median age of 26 years (range, 20–30); 83 were women and 47% of them were pregnant (Table 1). Among the 89 positive cultures, a single bacterium was isolated in 78, and 11 evidenced at least two different pathogenic bacteria. A total of 100 pathogenic bacteria were isolated. Ninety-four of the strains showed growth greater than 1 × 10^5^ colony-forming units per millilitre (CFU/mL).

The 100 microorganisms identified were as follows: *E. faecalis* (19), *E. faecium* (33)*, E. hirae* (5), *Escherichia coli* (28), *S. aureus* (1), *S. epidermidis* (3), *S. haemolyticus* (1), *S. hominis* (1), *Klebsiella pneumoniae* (2), *Enterobacter cloacae* (1), *Streptococcus* spp. *S. agalactiae* (2), *S. gallocyticus* (1), *Acinetobacter baumanii* (1), *A. junnii* (1), and *Pseudomonas putida* (1).

### 2.1. On-Site Susceptibility Testing

The results obtained for the antibiotic susceptibility test are shown in Table 2.

### 2.2. Resistance Values for Gram-Negative Bacteria

More than half of the antibiotics tested showed a resistance rate higher than 50% (Table 3). The oral antibiotics to which the bacteria showed the greatest susceptibility were fosfomycin (9.4%), and nitrofurantoin (12.9%).

Fourteen of the 34 Gram-negative bacteria were positive for ESBL.

### 2.3. Resistance Values for Gram-Positive Bacteria

The resistance rates for ciprofloxacin and levofloxacin were 27.3% for both drugs. The antibiotics with the lowest resistance rates were nitrofurantoin (16.3%), gentamicin in high concentration (14.8%), tygecicline (7.7%), teicoplanin (3.3%), and linezolid and vancomycin (1.7%) (Table 4). In the case of enterococcus, practically no strains resistant to teicoplanin, linezolid, or vancomycin were found.

### 2.4. Antimicrobial Profile for Main Species of Bacterial Isolates

*E. coli* isolates showed high resistance levels to betalactamics (amoxicillin-clavulanic acid (60%), ampicillin (88%)) and third-generation cephalosporines (ceftazimide (72%)) (Table 5).

*E. faecium* isolates presented good sensitivity to high concentration gentamycin (6%) and linezolid (0%). None of the isolates of E. faecium was resistant to vancomycin. 

*E. faecalis* isolates presented good sensitivity to nitrofurantoin, Fosfomycin (0%) and linezolid (5%). One of the isolates results to be resistant to vancomycin.

## 3. Discussion

The present study was conducted to determine the prevalence of UTIs and the bacterial agents involved in these infections, as well as to determine the drug susceptibility profile of the bacterial uropathogens implicated. Ninety percent of culture-confirmed UTIs were caused by three uropathogens, of which *E. coli* and *E. faecium* were the most common. In Uganda, suspected UTIs are treated empirically, though symptoms may be confounded with other causes such as pregnancy-related changes or other infections [13]. Only 134 patients (13.3%) showed leukocyturia, and bacterial growth was detected in 89 of them, indicating widespread, unnecessary use of antibiotics, leading to serious problems of bacterial resistance and therapeutic ineffectiveness. 

The finding that most strains isolated were enterococci (57%) contrasts sharply with results from similar studies carried out in Western Europe and the United States, where the incidence of urinary infections by enterococci ranges between 5% and 18% [14,15]. However, this result is much more similar to those obtained in other developing countries in Africa, Asia, or Eastern Europe, where the rate of UTIs caused by enterococci is between 20% and 60% [16,17,18]. This difference may be due to the poor sanitation and poor health care in rural areas when compared to African capitals and other large cities, which are more widely studied [18]. Moreover, the design of the study can also affect these results: during pregnancy, it is usual for women to experience physical changes in the genital tract, which may increase the risk of infection by Gram-positive bacteria [2].

The results of on-site antimicrobial susceptibility tests performed using disk diffusion in Uganda were consistent with those obtained for the same strains in Spain, which detected the minimum inhibitory concentration with the Phoenix M50 BD system (Table 2). For most of the antibiotics tested, the discrepancy between the two methods was less than 15%. The disk diffusion assay offers many advantages: simplicity, low cost, and the ease of interpreting the results provided. It also provides qualitative results by categorising bacteria as susceptible, intermediate, or resistant, which can be very useful in a laboratory where MIC cannot be performed. For this reason, we wanted to compare the quality of its results against an automated method that determines MIC. However, despite the use of quality controls, there are differences between the two methods compared for levofloxacin and ciprofloxacin. These may be due to the limited resources of the laboratory where the agar disk diffusion method was performed. Problems with the electrical supply or humidity control can alter both the incubation conditions of the microorganism and those of the conservation of the antibiogram discs. 

The susceptibility profiles of isolated pathogens demonstrate the high efficacy of nitrofurantoin, which has an in vitro susceptibility close to 90%. It should also be noted that pathogens with intrinsic resistance to nitrofurantoin, such as *Proteus* spp. or other bacteria with the urease enzyme, were absent. In most cases, these results are consistent with other studies conducted in Uganda and the rest of Africa [1,4,9,19]. 

Amoxicillin and ciprofloxacin, which are used as second-line treatment for UTI in Uganda [9], presented very high levels of resistance, and data from our study show that these two antibiotic drugs are totally unviable as a therapeutic alternative. In the case of ciprofloxacin, its resistance levels were even higher than those obtained in other studies in Uganda and West Africa [4,9,10,19]. 

We found high resistance levels to amoxicillin-clavulanic. Of 34 Gram-negative bacteria, 14 were ESBL producers. This prevalence is very high compared to other results obtained in similar studies in Africa [19], but in results obtained from 2015 to 2019, we can observe a fast increase in ESBL-producing bacteria.

Overall, the results of this study depict a worrisome scenario. The second line of treatment proposed by local governments may be totally ineffective given the high levels of amoxicillin and ciprofloxacin resistance shown here. These two antibiotics are currently more affordable than other drugs such as nitrofurantoin [20]. The easy, uncontrolled access to less effective antibiotics at local pharmacies, as well as the widespread failure to complete the treatment cycle, often due to the low income of the population and the sale of medication on a single-dose basis, pose a serious problem of antibiotic resistance.

## 4. Materials and Methods

### 4.1. Location

The study was carried out at Saint Joseph Kitgum Hospital, in the Kitgum region of Uganda. Located 450 km from the capital of the country, this rural area has 2 reference hospitals: one public (Kitgum General Hospital, with 200 beds) and another private (Hospital of Saint Joseph, with 350 beds). The latter, the site of our study, is the reference centre for health care in the regions of Kotido, Abim, Moroto, Agago, and Pader.

The hospital laboratory is equipped with basic materials (e.g., microscopes, autoclave, computer with internet connection, running water, and electricity), as well as technical staff trained in microscopic diagnosis (i.e., 2 laboratory technicians, 5 laboratory assistants, 1 cleaning assistant).

### 4.2. Inclusion Criteria

This prospective study included patients older than 16 years of age with leukocyturia and a urine concentration of over 125 leukocytes/mL [6] and symptoms compatible with a UTI, including suprapubic pain, burning sensation when urinating, incontinence, lower back pain, or patients in whom the presence of nitrites in urine was detected in routine urinalysis. We excluded all patients who had received empirical antibiotic treatment prior to the collection of the urine sample as well as all patients who underwent broad-spectrum antibiotic treatment for any condition within the 2 weeks before the collection of the sample.

### 4.3. Sample Collection and Processing

Urine samples were collected in accordance with the instructions of the laboratory staff, all of whom are bilingual in English and the Luo dialect. Samples were collected by patients after washing their hands. Sterile receptacles were filled with samples and labelled with the number assigned consecutively to the patient for the purposes of the study [7,8]. Men were instructed to retract the foreskin and then collect mid-stream urine, after about 2 s of urination. Women were requested to separate their labia and to clean them with sterile gauze impregnated with saline. The samples were immediately taken to the laboratory for analysis, no more than 30 min after collection [21].

A test of the reactive strip (Urine-10 Strips, 100 strips, Cypress Diagnostics, Belgium) was performed on all urine samples collected [8,10]. The levels of haemoglobin, glucose, proteins, nitrites, and pH were recorded. All samples were also subjected to microscopic analysis to measure the concentration of epithelial cells and white and red blood cells [22].

One-millilitre aliquots from the samples meeting the established criteria were divided and centrifuged at 3500 rpm for 5 min [13]. Subsequently, a Gram stain was performed directly from the sediment.

Ten microliters from each sample were inoculated using a seed handle on trypticase soy supplemented with 5% lamb blood agar (Biomérieux, Craponne, France) and incubated for at least 18 h in aerobiosis at 36 °C. After incubation, colony-forming units were counted, and their characteristics were studied.

Antimicrobial susceptibility studies were performed with a 0.5 McFarland inoculum in 0.9% NaCl saline on Mueller Hinton agar (Biomérieux, Craponne, France). The antibiotics tested were amoxicillin (25 µg), amoxicillin/clavulanic acid (20 + 10 µg), ciprofloxacin (5 µg), levofloxacin (5 µg), vancomycin (5 µg), trimethoprim/sulfamethoxazole (1.25 + 23.75 µg), nitrofurantoin (100 µg), gentamicin (15 µg), and ampicillin (10 µg). Oxacillin was also tested for Gram-positive bacteria.

The antimicrobial susceptibility for each of the antibiotics tested was determined by measuring the inhibition halo according to the EUCAST criteria [14]. Prepared 90 mm plates were used, with a maximum of 6 disks per plate.

All isolated bacterial strains were preserved in 1.5 mL tubes with reconstituted and self-induced powdered milk (Sarstedt, Numbrecht, Germany), and frozen at −20 °C for transportation to the laboratory in Spain. The isolated strains were analysed a second time in the Hospital Príncipe de Asturias (Alcalá de Henares, Madrid, Spain); these were plated on trypticase soy blood agar for identification by matrix-assisted laser desorption/ionisation time-of-flight (MALDI-TOF) spectrometry (BD Bruker MALDI Biotyper System, Franklin Lakes, NJ, USA).

Once the bacteria were identified, we determined the susceptibility profile of each strain using the Becton Dickinson Phoenix M50 device. Established antibiotic batteries were used for Gram-positive (P-502) and Gram-negative (P-96) UTIs. This device also recognises ESBL-producing bacteria due to their susceptibility to third- and fourth-generation cephalosporines. EUCAST breakpoints are used to categorise results into three susceptibility categories. (1) S—Susceptible, standard dosing regimen: A microorganism is categorised as Susceptible, standard dosing regimen, when there is a high likelihood of therapeutic success using a standard dosing regimen of the agent. (2) I—Susceptible, increased exposure: A microorganism is categorised as Susceptible, increased exposure*, when there is a high likelihood of therapeutic success because exposure to the agent is increased by adjusting the dosing regimen or by its concentration at the site of infection. (3) R—Resistant: A microorganism is categorised as Resistant when there is a high likelihood of therapeutic failure even when there is increased exposure.

*Exposure is a function of how the mode of administration, dose, dosing interval, infusion time, and the distribution and excretion of the antimicrobial agent will influence the infecting organism at the site of infection.

The IBM SPSS statistical package for Windows, version 22.0, was used (IBM Corp., Armonk, NY, USA). Continuous variables are expressed as medians and interquartile ranges, while qualitative variables appear as absolute and relative frequencies.

## 5. Conclusions

Enterococci and. *E. coli* were the most common etiological agents isolated in this region. The very high resistance levels to amoxicillin and ciprofloxacin, which are defined as second-line treatment in the 2016 Ugandan Clinical Guidelines, indicate that these protocols do not reflect the current situation. This large discrepancy between the empirical treatments administered and the sensitivity profiles of the main etiological agents reveals the need to update these protocols. The number of ESBL-producing Gram-negative bacteria found is highly disturbing. We recommend performing clinical microbiology studies such as urine culture and antibiograms in patients undergoing UTI treatment until empirical treatment guides are in sync with the sensitivity profiles found in this study.

## Figures and Tables

**Table 1:** Clinical and demographic characteristics of the patients included in the study.

	Men (6)		Women (83)	
Age (median, IQR)	26 (20–30)		24 (21–30)	
Pregnant during infection	-	-	39	47%

**Table 2:** Comparison of antibiotic susceptibility profiles obtained for the same strains between on-site testing in a rural hospital in Uganda (disk-plate method) and at Príncipe de Asturias hospital in Alcalá de Henares, Spain (Phoenix M50 BD Device).

Antibiotic (*n*)	Resistance Determined by Disk-Plate Method (%)	Resistance Determined by BD-Phoenix M50 (%)	Difference
Amoxicillin-clavulanic acid (18)	7 (38.9)	10 (56)	16.7%
Ampicillin (28)	14 (50)	12 (43)	7.1%
Oxacillin (22)	21 (95)	21 (95)	0%
Ciprofloxacin (28)	23 (82.1)	10 (35.7)	46.5%
Levofloxacin (30)	17 (57)	8 (27)	30%
Gentamycin (29)	17 (58.2)	21 (72)	13.8%
Vancomycin (21)	2 (9.5)	1 (4.7)	4.8%
Sulfamethoxazole-trimethoprim (49)	47 (96)	46 (94)	2%
Nitrofurantoin (24)	3 (12.5)	4 (17)	4.5%

**Table 3:** Antimicrobial sensitivity profile of Gram-negative bacteria obtained using the MD Phoenix M50 device. S—Susceptible, standard dosing regimen. I—Susceptible, increased exposure. R—Resistant: A microorganism is categorised as Resistant when there is a high likelihood of therapeutic failure even when there is increased exposure.

Gram-Negative Bacteria (*n*, Uganda)	R (%)	I (%)	S (%)
Amoxicillin/clavulanic acid (34)	22 (64.7)	0 (0)	12 (35.3)
Ampicillin (32)	29 (90.60)	0 (0)	3 (9.4)
Piperacillin (32)	28 (87.5)	0 (0)	4 (12.5)
Piperacillin-tazobactam (32)	8 (25)	2 (6.25)	22 (68.75)
Mecillinam (31)	14 (45.20)	1 (3.2)	16 (51.6)
Cefepime (33)	24 (72.7)	0 (0)	9 (27.3)
Cefixime (33)	23 (69.7)	1 (3)	9 (27.3)
Ceftazidime (31)	22 (71)	0 (0)	9 (29)
Ceftazidime-avibactam (32)	21 (65.6)	2 (6.25)	9 (28.15)
Ceftriaxone (31)	2 (6.5)	0 (0)	29 (93.5)
Cefuroxime (31)	21 (67.7)	0 (0)	10 (32.3)
Cefalexin (34)	25 (73.5)	0 (0)	9 (26.5)
Ertapenem (31)	2 (6.5)	0 (0)	29 (93.5)
Imipenem (34)	1 (2.9)	1 (2.9)	32 (94.2)
Meropenem (33)	2 (6.1)	1 (3)	30 (90.9)
Aztreonam (34)	23 (67.6)	0 (0)	11 (32.4)
Ciprofloxacin (34)	20 (58.8)	3 (8.8)	11 (32.4)
Levofloxacin (34)	19 (55.9)	0 (0)	15 (44.1)
Gentamicin (33)	11 (33.3)	0 (0)	22 (66.7)
Tobramycin (34)	15 (44.1)	0 (0)	19 (55.9)
Tigecycline (31)	1 (3.2)	1 (3.2)	29 (93.6)
Fosfomycin (32)	3 (9.4)	0 (0)	29 (90.6)
Nitrofurantoin (31)	4 (12.9)	0 (0)	27 (87.1)
Trimethoprim/sulfamethoxazole (33)	30 (90.9)	0 (0)	3 (9.1)

**Table 4:** Gram-positive bacteria sensitivity profile obtained using the MD Phoenix M50 BD device. S—Susceptible, standard dosing regimen. I—Susceptible, increased exposure. R—Resistant: A microorganism is categorised as Resistant when there is a high likelihood of therapeutic failure even when there is increased exposure.

Gram-Positive Bacteria (*n*)	R (%)	I (%)	S (%)
Penicillin G (39)	39 (100)	0 (0)	0 (0)
Ampicillin (60)	17 (28.3)	0 (0)	43 (71.3)
Oxacillin (66)	64 (97)	0 (0)	2 (3)
Ceftaroline (51)	49 (96.1)	0 (0)	2 (3.9)
Cefoxitin (47)	47 (100)	0 (0)	0 (0)
Imipenem (55)	14 (25.5)	0 (0)	41 (74.5)
Ciprofloxacin (55)	15 (27.3)	0 (0)	40 (72.7)
Levofloxacin (55)	15 (27.3)	0 (0)	40 (72.7)
Gentamycin (60)	52 (86.7)	0 (0)	8 (13.3)
High concentration gentamycin (54)	8 (14.8)	0 (0)	46 (85.2)
Tobramycin (55)	52 (94.5)	0 (0)	3 (5.5)
Kanamycin (51)	51 (100)	0 (0)	0 (0)
Teicoplanin (60)	2 (3.3)	0 (0)	58 (96.7)
Vancomycin (60)	1 (1.7)	0 (0)	59 (98.3)
Clindamycin (25)	25 (100)	0 (0)	0 (0)
Erythromycin (55)	55 (100)	0 (0)	0 (0)
Quinupristin/dalfopristin (55)	26 (47.3)	15 (27.3)	14 (25.4)
Tigecycline (26)	2 (7.7)	0 (0)	24 (92.3)
Linezolid (60)	1 (1.7)	0 (0)	59 (98.3)
Fusidic acid (55)	49 (89.1)	0 (0)	6 (10.9)
Nitrofurantoin (49)	8 (16.3)	0 (0)	41 (83.7)
Trimethoprim/sulfamethoxazole (60)	58 (96.7)	0 (0)	2 (3.3)
Trimethoprim (60)	60 (100)	0 (0)	0 (0)

**Table 5:** Antimicrobial profile for main species of bacterial isolates obtained using the Phoenix M50 BD device. R—Resistant: A microorganism is categorised as Resistant when there is a high likelihood of therapeutic failure even when there is increased exposure.

Antibiotic	*E. coli* (*n* = 25)	*E. faecium* (33)	*E. faecalis* (19)
Amoxicillin/clavulanic acid	15 * (60.0%)	-	-
Ampicillin	22 (88.0%)	10 (30.3%)	0 (0%)
Penicillin G	-	30 (90.9%)	-
Piperacillin	25 (100%)	-	-
Piperacillin-tazobactam	5 (20.0%)	-	-
Mecillinam	11 (44.0%)	-	-
Cefepime	18 (72.0%)	-	-
Cefixime	17 (68.0%)	-	-
Ceftazidime	18 (72.0%)	-	-
Ceftazidime-avibactam	16 (64.0%)	-	-
Ceftriaxone	2 (8.0%)	-	-
Cefuroxime	17 (68.0%)	-	-
Ceftarolyne	-	30 (90.9%)	19 (100%)
Cefoxitin	-	29 (87.9%)	19 (100%)
Cefalexin	18 (72.0%)	-	-
Ertapenem	2 (8.0%)	-	-
Imipenem	0 (0%)	10 (30.3%)	0 (0%)
Meropenem	1 (4.0%)	-	-
Aztreonam	16 (64.0%)	-	-
Ciprofloxacin	14 (56.0%)	9 (27.3%)	4 (21.1%)
Levofloxacin	14 (56.0%)	8 (24.4%)	4 (21.1%)
Gentamicin	6 (24.0%)	30 (90.9%)	19 (100%)
High concentration Gentamycin	-	2 (6%)	5 (26.3%)
Kanamycin	-	30 (90.9%)	19 (100%)
Teicoplanin	-	1 (3%)	1 (5%)
Vancomycin	-	0 (0%)	1 (5%)
Clindamycin	-	-	19 (100%)
Erythromycin	-	30 (90.9%)	19 (100%)
Quinupristine/Dalfopirstine	-	7 (21.2%)	19 (100%)
Linezolid	-	0 (0%)	1 (5%)
Fusidic acid	-	29 (87.9%)	19 (100%)
Trimetoprim	-	29 87.9%)	19 (100%)
Tobramycin	11 (44.0%)	30 (90.9%)	19 (100%)
Tigecycline	1 (4.0%)	-	0 (0%)
Fosfomycin	2 (8%)	-	0 (0%)
Nitrofurantoin	1 (4%)	8 (24.2%)	0 (0%)
Trimethoprim/sulfamethoxazole	23 (92%)	30 (90.9%)	19 (100%)

* R%.

## Data Availability

Not applicable.
